# Diagnosis of spontaneous secondary tension pneumothorax following apparent recovery from coronavirus disease 2019 pneumonitis: a case report

**DOI:** 10.1186/s13256-022-03313-x

**Published:** 2022-02-22

**Authors:** Romesh Tirimanna, James Myerson, Michael Okorie, Eleanor Dorman

**Affiliations:** 1 Brighton and Sussex University Hospitals NHS Trust, Brighton, UK; 2 Respiratory Medicine, Brighton and Sussex University Hospitals NHS Trust, Brighton, UK; 3 Clinical Pharmacology, Brighton and Sussex University Hospitals NHS Trust, Brighton, UK; 4 Barking Havering and Redbridge Hospitals NHS Trust, London, UK

**Keywords:** COVID-19, Tension, Pneumothorax, Emergency, Thoracostomy, Case report

## Abstract

**Background:**

Coronavirus disease 2019 has been associated with a plethora of different manifestations of systems affected (including pulmonary, gastrointestinal, and thrombotic disease) and time to presentation of complications. Pneumothorax has been established as a complication in the literature. However, tension pneumothorax remains a rare presentation with higher mortality. We report a case of secondary tension pneumothorax in a patient following apparent recovery from coronavirus disease 2019 pneumonitis.

**Case presentation:**

Eight days after resolution of coronavirus disease 2019 pneumonitis symptoms, a 51-year-old Caucasian man with no pre-existing pulmonary disease was brought into the emergency department following 48 hours of progressive shortness of breath. Further clinical assessment revealed reduced breath sounds in the right lung, blood pressure was 116/95 mmHg, and jugular venous pressure was not elevated. Chest x-ray showed right-sided tension pneumothorax with mediastinal shift. Insertion of a chest drain led to rapid resolution of symptoms, and the patient was discharged following full re-expansion of the lung.

**Conclusions:**

The period of recovery from coronavirus disease 2019 is variable. Clinicians should consider tension pneumothorax as a possible complication of coronavirus disease 2019 pneumonitis in patients presenting with type 1 respiratory failure, even after resolution of pneumonitis symptoms and a considerable time period following initial contraction of coronavirus disease 2019.

## Background

The coronavirus disease 2019 (COVID-19) pandemic remains a relatively recent phenomenon with various long-term sequelae still being identified. In susceptible individuals, infection with severe acute respiratory syndrome coronavirus 2 (SARS-CoV-2) can trigger a profound inflammatory state resulting in a rapidly progressing pneumonitis and ground glass opacification on chest x-ray [1]. COVID-19 pneumonitis is treated with oxygen and corticosteroids in addition to other forms of pharmacological therapy.

This patient was admitted at a time when daily case numbers for COVID-19 exceeded 40,000 and daily deaths exceeded 900 in the UK. The ubiquity of COVID-19 pneumonitis risked causing tunnel vision among many clinicians at the time. Tension pneumothorax, by contrast, is a relatively uncommon presentation with high mortality. One systematic review estimated the mortality of tension pneumothorax at 6.7% in unventilated patients, increasing up to 22.7% in ventilated patients [2]. This case report aims to emphasize the importance of recognizing tension pneumothorax as a possible complication of COVID-19 pneumonitis even after apparent recovery.

## Case presentation

A 51-year-old Caucasian male gardener initially presented to the emergency department (ED) following a 4-day history of shortness of breath, dry cough, and weakness. He had a positive lateral flow test for SARS-CoV-2, and chest x-ray revealed bilateral patchy opacification. He maintained oxygen saturation above 94% on room air so returned home with an oxygen saturation monitor.

**Fig. 1:**
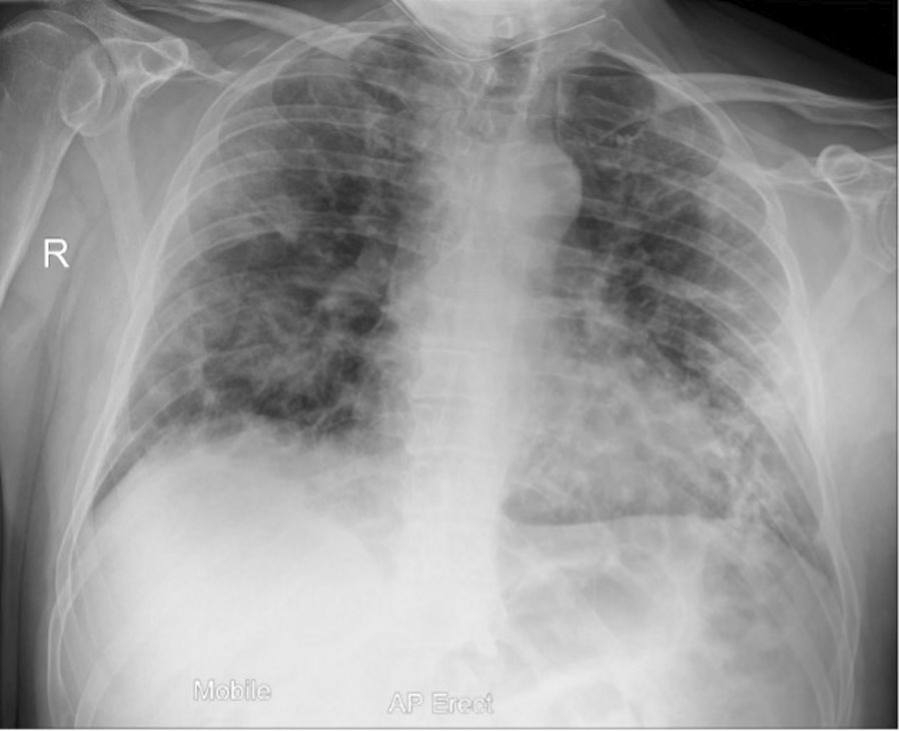
Chest x-ray on initial admission to hospital: Extensive multifocal opacities throughout lungs in keeping with COVID-19 pneumonitis

Twelve days after onset of symptoms, the patient re-presented to the ED, reporting that his oxygen saturation had dropped to 78%. His chest x-ray showed significant progression of COVID-19 pneumonitis (Fig. 1) in comparison with the initial chest x-ray. He was admitted and received oxygen therapy, dexamethasone, doxycycline, and amoxicillin. The antibiotics were later stopped in view of the patient’s low procalcitonin (< 0.25) and remaining afebrile. The failure to wean his oxygen and raised d-dimer of 20.0 triggered a computed tomography pulmonary angiogram (CTPA), which ruled out pulmonary embolism (PE). After 3 days as a ward inpatient, he was again able to maintain satisfactory oxygen saturation on room air with a significant reduction in inflammatory markers. He was discharged to complete a 10-day course of dexamethasone.

**Fig. 2:**
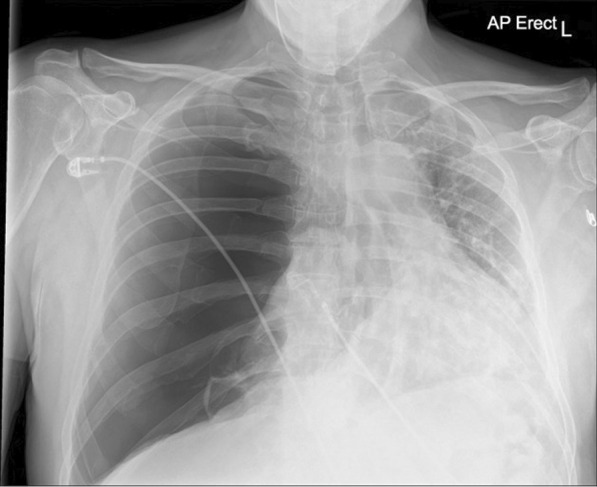
Chest x-ray of tension pneumothorax: Large right-sided tension pneumothorax causing deviation of the mediastinum to the left. The left lung is compressed

**Fig. 3:**
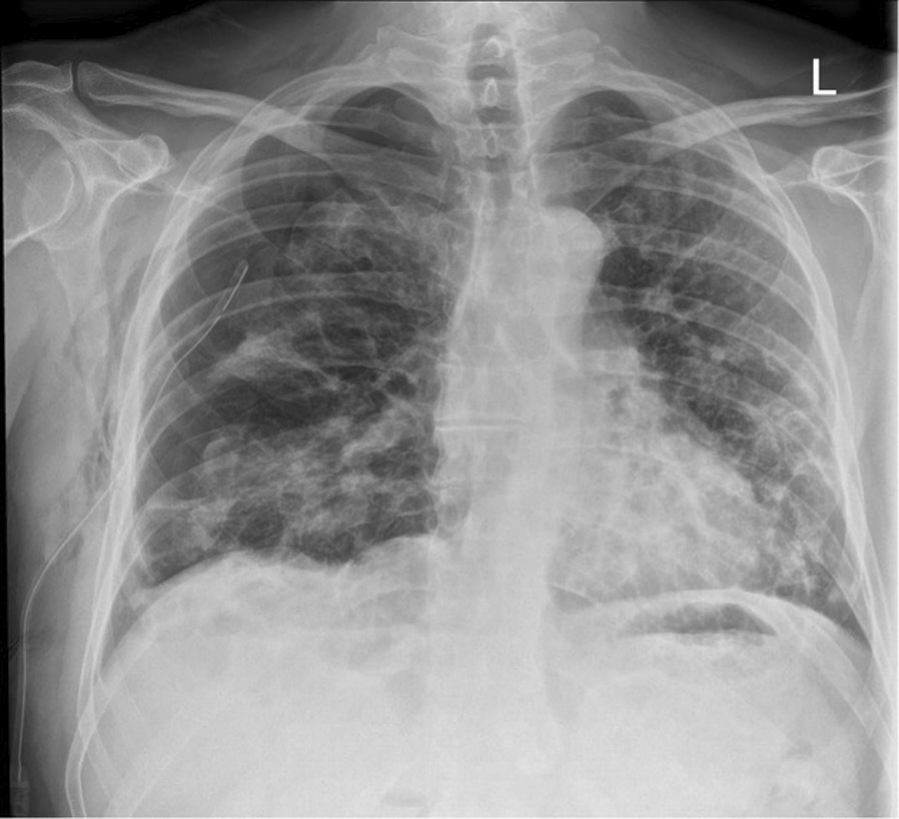
Chest x-ray of tension pneumothorax following chest drain insertion: Partial resolution of tension pneumothorax

**Fig. 4:**
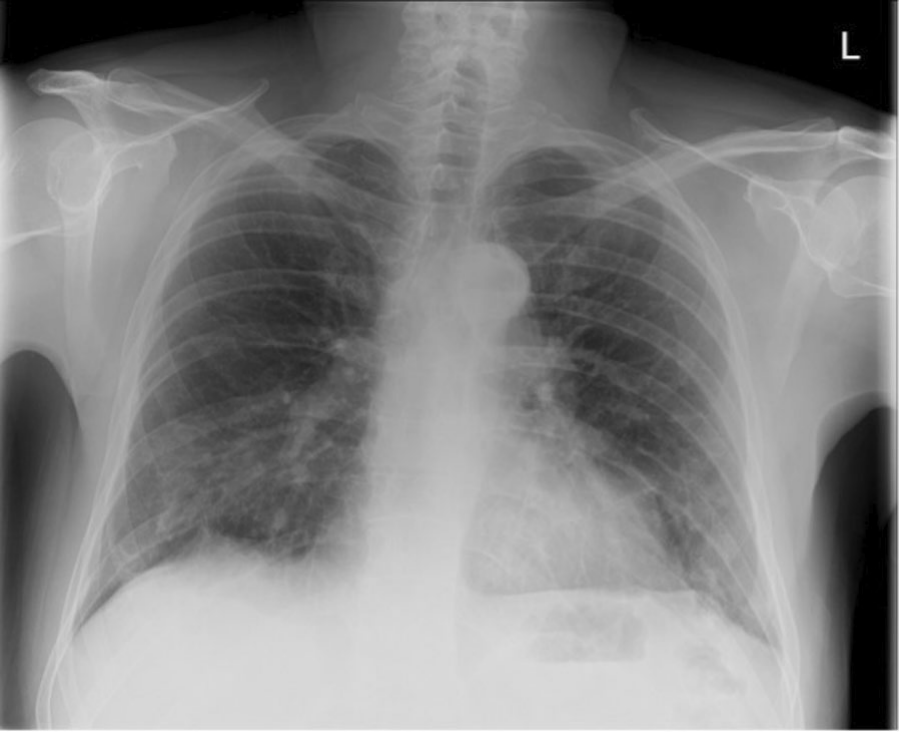
Chest X-ray on outpatient follow-up 1 month after discharge

Twenty-three days after onset of symptoms (8 days after discharge from hospital), the patient experienced a 48-hour period of progressively worsening shortness of breath. He re-presented to the ED, and on examination reduced breath sounds and reduced chest expansion were noted on the right. The patient had a 4 L/min oxygen requirement. He was alert, tachypnoeic (40 breaths/minute), and tachycardic (129 beats/minute), with maintaining blood pressure (116/94 mmHg). Arterial blood gas analysis showed respiratory alkalosis (pH 7.53, PaO_2_ 8.3, PCO_2_ 3.6, HCO_3_ 22.6). The absence of typical clinical signs of tension pneumothorax (hemodynamic instability, tracheal deviation, and raised jugular venous pressure (JVP)) prompted investigation with chest x-ray rather than immediate needle decompression. His chest x-ray showed right-sided tension pneumothorax (Fig. 2), which was managed by insertion of 12F pigtail drain using Seldinger technique. The chest drain remained *in situ* for 4 days before chest x-ray showed adequate resolution of pneumothorax to allow for removal (Fig. 3). The patient was discharged following full re-expansion of the lung and resolution of symptoms 5 days post admission. The patient had a follow-up chest x-ray 1 month after discharge, which showed complete resolution of the pneumothorax (Fig. 4).

His only comorbidity of note was diet-controlled type II diabetes mellitus (HbA1C 64). He had no history of previous trauma, pulmonary disease, or thoracic surgery. There was no reported exposure to asbestos or other hazardous occupational chemicals, and the patient reported no recent activities associated with risk of barotrauma. He was a nonsmoker, independent in all activities of daily living, and had good exercise tolerance. There was no family history of connective tissue diseases or malignancy.

## Discussion and conclusions

This patient presented 23 days from his initial symptoms of COVID-19 and was thought to have recovered from COVID-19 pneumonitis; disease progression was deemed unlikely. The overwhelming prevalence of COVID-19 coupled with relatively rudimentary understanding of the disease at the time meant that this presentation could easily be subject to diagnostic overshadowing.

A key differential to consider for acutely deteriorating patients in type 1 respiratory failure is PE. COVID-19 infection causes a hypercoagulable state which has been associated with increased risk of venous thromboembolism (VTE) [3, 4]. During this patient’s first admission, in view of his raised d-dimer and oxygen requirement, a CTPA was performed, which excluded PE.

Pneumothorax has been reported as a complication of COVID-19 infection by Martinelli *et al.* This case series included 60 patients with COVID-19 who developed or presented with pneumothoraces [5]. Tension pneumothorax differs in presentation, causing hemodynamic compromise, tracheal deviation, and raised JVP [6]. The patient we discuss did not have these typical signs, but likely had physiological reserve to compensate, resulting in diagnostic uncertainty.

Vahidirad *et al*. describe a similar case where a patient was admitted with COVID-19 pneumonitis and went on to develop tension pneumothorax 20 days after onset of symptoms [7]. This patient received a different treatment regime during their 5-day hospital admission, including azithromycin, lopinavir/ritonavir (KALETRA), and low-dose dexamethasone. Spiro *et al*. reported a case of a 47-year-old male patient who had tension pneumothorax diagnosed on CT scan 11 days following first admission [8]. Flower *et al*. reported a 36-year-old male patient who presented to A&E with tension pneumothorax seen on X-ray 2–3 weeks from onset of symptoms [9]. The cases also did not present with typical signs of tension pneumothorax.

Tension pneumothorax results from a defect in the pleura that allows one-way air entry into the pleural space. A pleural defect can occur due to rupture of large bullae, alveolar rupture, or from connection to pneumomediastinum [10]. This causes the intrapleural pressures to increase, exceeding intra-alveolar pressure and leading to lung collapse and mediastinal shift.

The etiology of tension pneumothorax can be subdivided into primary or secondary, with secondary tension pneumothorax developing because of underlying pulmonary disease. We consider this patient to have secondary tension pneumothorax following COVID-19 pneumonitis.

Needle thoracostomy is the recommended management of tension pneumothorax in advanced trauma life support (ATLS) guidelines. This requires confidence in the clinical diagnosis, surface anatomy, and procedural skills. There are several reports of ineffective needle decompression with no resolution of symptoms until chest drain insertion [11]. Non-operator factors attributed to unsuccessful needle decompression include inadequate length and kinking of the canula.

Leigh-Smith *et al*. conducted a systematic review that concluded that immediate chest drain insertion is preferred to needle thoracostomy in awake non-ventilated patients without hemodynamic instability. The patient we discuss was not in extremis, so management with chest drain was deemed appropriate.

The pathophysiology of tension pneumothorax secondary to COVID-19 infection is not described in the literature. Factors that may be of significance include reduced lung compliance, pleural inflammation, mechanical ventilation, and pre-existing pulmonary disease. Lung biopsy specimens of COVID-19 patients showed alveolar swelling with inflammation of the alveolar membrane [12]. This can progress to acute respiratory distress syndrome (ARDS) where pneumocyte destruction is seen. Pneumothorax occurs more commonly in patients with reduced lung compliance, especially when mechanically ventilated [13]. Grasseli *et al*. found that lung compliance in COVID-19-related ARDS was 28% higher compared with those with ARDS unrelated to COVID-19 [14]. Therefore, one may expect a lower incidence of tension pneumothorax in COVID-19-related compared with classical ARDS.

In patients with subacute presentation of tension pneumothorax, accurate and timely diagnosis is key. Point of care ultrasound (POCUS) is a bedside scan available in the ED that has higher sensitivity for diagnosis of pneumothorax when compared with AP chest x-ray and saves time, possibly preventing progression to hemodynamic instability [15]. Although diagnosis on clinical examination remains the gold standard for tension pneumothorax, POCUS could be considered instead of chest x-ray as initial investigation in diagnostic uncertainty.

A dearth of reports of COVID-19-related tension pneumothoraces means that there is a lack of data on incidence and mortality. Tension pneumothorax is an important differential for type 1 respiratory failure that should be considered in all patients with active or recently resolved COVID-19 pneumonitis and after a variable time period from their initial COVID-19 diagnosis.

## Data Availability

Data sharing is not applicable to this article as no datasets were generated or analyzed during the current study.

## Abbreviations

SARS-CoV-2: Severe acute respiratory syndrome coronavirus 2 disease
JVP: Jugular venous pressure
COVID-19: Coronavirus disease 2019
ED: Emergency department
PE: Pulmonary embolism
DVT: Deep venous thrombosis
VTE: Venous thromboembolism
CTPA: CT pulmonary angiogram
ATLS: Advanced trauma life support
ARDS: Acute respiratory distress syndrome
POCUS: Point of care ultrasound
